# FTIR-ATR-based prediction and modelling of lignin and energy contents reveals independent intra-specific variation of these traits in bioenergy poplars

**DOI:** 10.1186/1746-4811-7-9

**Published:** 2011-04-10

**Authors:** Guanwu Zhou, Gail Taylor, Andrea Polle

**Affiliations:** 1 Forest Botany and Tree Physiology, Büsgen Institute, Georg August University Göttingen, Büsgenweg 2, 37077 Göttingen, Germany; 2 Research Institute of Wood Industry, Chinese Academy of Forestry, 100091 Beijing, China; 3 School of Biological Sciences, University of Southampton, Bassett Crescent East SO16 7PX, UK

**Keywords:** Bioenergy, heat value, intraspecific variation, lignin, high throughput method, FTIR spectroscopy

## Abstract

**Background:**

There is an increasing demand for renewable resources to replace fossil fuels. However, different applications such as the production of secondary biofuels or combustion for energy production require different wood properties. Therefore, high-throughput methods are needed for rapid screening of wood in large scale samples, e.g., to evaluate the outcome of tree breeding or genetic engineering. In this study, we investigated the intra-specific variability of lignin and energy contents in extractive-free wood of hybrid poplar progenies (*Populus trichocarpa × deltoides*) and tested if the range was sufficient for the development of quantitative prediction models based on Fourier transform infrared spectroscopy (FTIR). Since lignin is a major energy-bearing compound, we expected that the energy content of wood would be positively correlated with the lignin content.

**Results:**

Lignin contents of extractive-free poplar wood samples determined by the acetyl bromide method ranged from 23.4% to 32.1%, and the calorific values measured with a combustion calorimeter varied from 17260 to 19767 J g^-1^. For the development of calibration models partial least square regression and cross validation was applied to correlate FTIR spectra determined with an attenuated total reflectance (ATR) unit to measured values of lignin or energy contents. The best models with high coefficients of determination (R^2 ^(calibration) = 0.91 and 0.90; R^2 ^(cross-validation) = 0.81 and 0.79) and low root mean square errors of cross validation (RMSECV = 0.77% and 62 J g^-1^) for lignin and energy determination, respectively, were obtained after data pre-processing and automatic wavenumber restriction. The calibration models were validated by analyses of independent sets of wood samples yielding R^2 ^= 0.88 and 0.86 for lignin and energy contents, respectively.

**Conclusions:**

These results show that FTIR-ATR spectroscopy is suitable as a high-throughput method for lignin and energy estimations in large data sets. Our study revealed that the intra-specific variations in lignin and energy contents were unrelated to each other and that the lignin content, therefore, was no predictor of the energy content. Employing principle component analyses we showed that factor loadings for the energy content were mainly associated with carbohydrate ring vibrations, whereas those for lignin were mainly related to aromatic compounds. Therefore, our analysis suggests that it may be possible to optimize the energy content of trees without concomitant increase in lignin.

## Background

There is an increasing demand for the production of fast-growing woody plants such as poplars as a sustainable resource for the production of biofuels, heat or electricity as well as for pulp and paper production. However, efficient wood utilization is strongly affected by the lignification of the cell wall. Lignin is an intensely cross-linked heteropolymer that renders plant cell walls rigid, hydrophobic and chemically stable; in angiosperms lignin is mainly composed of guaiacyl and syringyl units [1]. Wood processing requiring delignification is often an expensive bottleneck for its utilization. The lignin content is, therefore, a key target for breeding or genetic engineering to enhance wood properties [2]. The energy content of wood is another important breeding target because renewable biomass feedstock is increasingly used to replace fossil fuels in modern heating systems operated with wood pellets.

Attempts to improve wood properties have mainly focused on poplars (*Populus *spp.) because these tree species display fast growth rates, can be grown in coppicing systems and are amenable to genetic modification [3,4]. The direct determination of lignin content by wet chemical methods and the measurement of energy contents are laborious and time-consuming. Hence reliable, high-throughput methods for the determination of lignin and the energy content are in great demand to identify valuable germplasm for specific applications such as bioenergy poplars for the conversion into bioethanol or for the production of heat and electricity.

Several spectroscopic techniques have been employed to analyze wood. For example, the chemical composition [5-8], basic density [9], and physical properties [10] of wood samples have been predicted by near infrared reflectance spectroscopy (NIRS) and multivariate statistical analyses. Furthermore, Fourier transform infrared (FTIR) spectroscopy has been applied for determination of wood density [11], chemical composition [12-14], lignin distribution [15], discrimination of wood from various tree species [16,17], and changes in wood properties during wood composites manufacture [18,19]. NIRS uses infrared light to detect overtones and combinational vibrations, whereas FTIR employs mid infrared regions of the radiation to detect primarily functional and fundamental vibrations of the molecular constituents of the irradiated matter. The FTIR absorption bands are often overlapping and make direct assignment of peaks to chemical constituents difficult [20]. Nevertheless, selection of wavenumbers related to lignin was successfully applied to predict lignin in eucalypt and spruce wood [21,22].

In the past decade, FTIR spectroscopy was improved by the development of an attenuated total reflectance (ATR) unit. Earlier measurements required preparation of samples in transparent KBr pellets whose absorbance was measured by the FTIR spectrometer [20]. In modern FTIR instruments an ATR crystal, which is pressed onto the untreated sample, enables direct interaction of the measuring beam with the sample and reflection of the attenuated radiation to the spectrometer. This technological advance increases the sensitivity of FTIR-based analyses and has, e.g., been used to determine the S/G ratio of poplar wood [7]. Calibrations to determine the lignin content of wood without preparation of KBr pellets have not yet been published. Furthermore, it may be possible to use the same spectra for the prediction of other wood properties. Here, we tested if FTIR-based models can be developed as tools for rapid prediction of the energy content.

The goal of the present study was to investigate the natural variability of lignin and energy contents in wood of field-grown poplar progenies of *Populus trichocarpa × P. deltoides *and to develop FTIR-based calibration models for high-throughput measurements of these traits. For this purpose the lignin and energy contents were determined in coppiced wood of the hybrid poplars with a modified acetyl bromide lignin assay and a calorimeter combustion test, respectively. FTIR-ATR spectra were recorded for extractive-free wood powder. Multivariate statistical analyses, in particular partial least square regression (PLSR) modelling, were applied to calibrating the FTIR spectra against the primary wet laboratory chemical data and the measured energy contents. Data pre-processing methods and automated selection of wavenumber ranges resulted in a high predictability and precise estimation of lignin contents and the calorific values of poplar wood from short-rotation forestry.

## Results and Discussion

### Natural variability in lignin and energy contents of poplar wood samples

**Figure 1:**
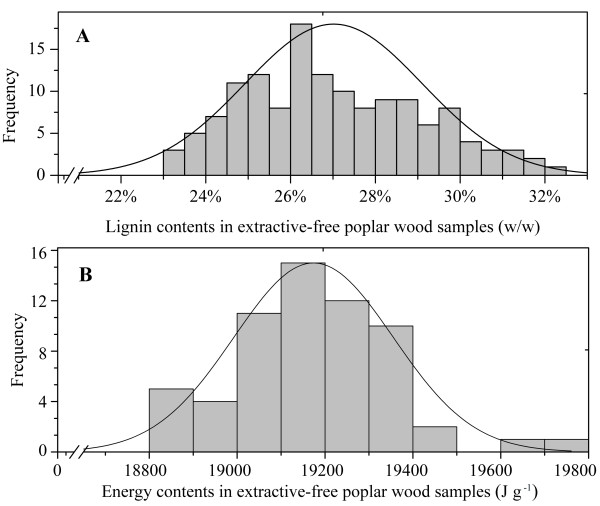
**Frequency distribution of lignin (A) and energy (B) content in extractive-free wood samples of hybrid poplar**.

The lignin contents of extractive-free wood samples of *P. trichocarpa × deltoides *ranged from 23.4% to 32.1% (w/w) with a mean of 27.0% (Figure 1A). The natural variation in lignin contents within this poplar plantation was comparable to that found for *Eucalyptus globulus *wood (23.4% - 34.5%, [21]) or for juvenile wood of Sitka spruce [22]. The different wood samples analysed here also broadly cover the variability observed for the lignin contents of different poplar species [23,24]. The natural variability in lignin in our sample set was an important precondition for the development of calibration models (see below), because if the range was too narrow, i.e., within one order of magnitude of the measurement error, it would be impossible to determine correlations between the FTIR-ATR measurements and the lignin contents.

The energy content in the extractive-free poplar wood samples ranged from 17260 to 19767 J g^-1 ^with a mean of 18514 J g^-1 ^(Figure 1B). Similar calorific values have previously been found in *P. × euramericana *wood from a short rotation plantation (average calorific value: 19.3 MJ kg^-1^; [15]), whereas straw of agricultural crops generally contains lower energy contents (*Glycine max *(L.) Merr.: 17.0 MJ kg^-1^, *Sorghum bicolor *(L.) Moench: 17.2 MJ kg^-1^, [25]).

Since lignin has about twice the energy content of cellulose [26], we wondered if the lignin content of the samples would correlate with their energy content. However, we obtained no significant linear correlation between the calorific values and lignin contents (R^2 ^= 0.0973, P-value = 0.1344). This contrasts previous reports in which strong correlations were obtained for these traits [27-29]. In contrast to our study, the previous analyses were conducted across different plant species, thus, encompassing a broader range of lignin and calorific values. Furthermore, untreated samples were used and therefore, additional constituents such as wood extractives may have affected the calorific values of biomass. Our study shows that the intra-specific variations in lignin and energy contents were unrelated in poplar.

### Analyses of FTIR-ATR spectra for the identification of chemical components contributing to the lignin and energy content of wood

**Figure 2:**
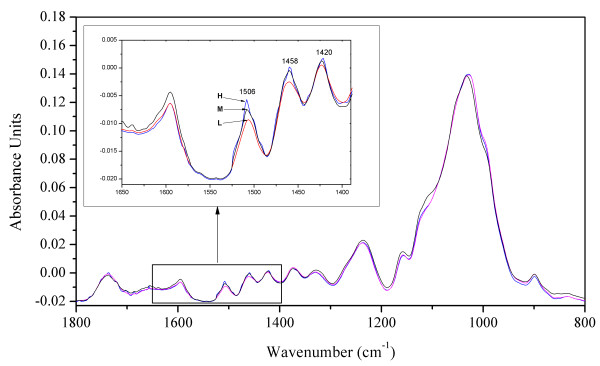
**FTIR-ATR spectra of wood samples with low (L), medium (M) and high (H) lignin contents in the finger-print region**. Each spectrum is the mean of three replicate samples. ATR-FTIR spectra were converted to transmission spectra by automatic correction for the wavenumber-dependent influence on the penetration depth on the radiation, then base-line corrected (Rubberband method) and pre-processed with the method of vector normalization. The lignin contents determined with the acetyl bromide method of the wood samples were L: 23.4%, M: 27.5% and H: 31.5%, respectively.

Figure 2 illustrates FTIR-ATR spectra of hybrid poplar coppice wood for the fingerprint region between 1800 cm^-1 ^and 800 cm^-1^. The absorption peaks were assigned tentatively to chemical components according to literature data [30-39]. The positions of the most characteristic bands for lignin in the fingerprint region are 1593 and 1506 cm^-1 ^for aromatic skeletal vibrations, 1458 and 1420 cm^-1 ^for C-H deformation, 1328 cm^-1 ^for syringyl ring plus guaiacyl ring, 1234 cm^-1 ^for syringyl ring and C = O stretch, and 1120 cm^-1 ^for aromatic skeletal vibrations (Figure 2). As shown in the inset a closer examination of the region from 1650 cm^-1 ^to 1380 cm^-1 ^indicated a clear association between absorbance changes in this region and differences in chemically determined lignin contents (Figure 2 inset). Therefore, this region was used for manual wavenumber selection during the cross validation phase (see below).

In contrast to lignin, nothing is known about wavenumber regions related to differences in wood energy contents. A direct comparison of FTIR spectra of wood samples with low, medium and high calorific values did not reveal any conspicuous absorption bands (not shown). To obtain evidence for the wood constituents that might be responsible for differences in energy content and those important for differences in lignin contents, we conducted principle component analyses (PCA) on two sets of selected samples: one consisting of spectra of samples with the lowest (10 samples), medium (10 samples) and highest energy contents (10 samples) according to their calorific values and the other of spectra of samples with lowest (10 samples), medium (10 samples) and highest (10 samples) lignin contents as determined by wet chemical analyses. Factor loadings of the two spectral sample sets were calculated in the wavenumber region from 1800 cm^-1 ^to 900 cm^-1 ^to identify the most divergent wavenumbers (Additional file 1, Figure S1, Additional file 2 Table S1). PC1, PC2, PC3 and PC4 of the "energy set" explained 73.3%, 20.1%, 5.5%, and 1.1% of the variation, respectively. In the "lignin sample set", the factor loadings of the first, second, third, and fourth PC explained 68.7%, 22.2%, 6.8%, and 2.3% of the variation respectively. The first three peaks in PC1 in the two data sets for energy and lignin samples were overlapping encompassing the carbohydrate region and a wavenumber for guaiacyl lignin (Additional file 1, Figure S1, Additional file 2, Table S1 and Additional file 3, Table S2). All other major peaks of the first four PCs diverged between the energy and lignin sets, respectively. Influential wavenumbers for wood energy content were identified mainly as peaks in the factor loadings for ring vibrations of carbohydrates (Additional file 2, Table S1). As expected, wavenumbers typical for aromatic compounds were prevalent in PCs for lignin (Additional file 3, Table S2: 14 out of 32 most divergent wavenumbers) but not in those for energy content (Additional file 2, Table S1: 7 out of 32). In conclusion, this analysis shows that the lack of correlation between lignin and energy content was the result of different constituents contributing either to energy content (mainly certain properties of the carbohydrates) or lignin (aromatic compounds), respectively.

### Predictive models for the estimation of lignin and energy content with FTIR-ATR spectroscopy

Quality spectra with high peak resolution and smoothness of baseline are a prerequisite for further quantitative analysis. As demonstrated by Faix and Böttcher [40], the traditional KBr pellet method suffers from poor spectral reproducibility caused by various factors including, among others, moisture content in the pellets, room humidity, sample inhomogeneity in the pellet, and variable pellet thickness. In contrast, wood powder can directly be used for FTIR-ATR spectroscopy. For our wood samples the reproducibility of the FTIR-ATR spectra was high (SD = 0.23%), which is a precondition for PLS prediction model building. Furthermore, analyses of the score plots of the PCA up to four factors did not reveal obvious patterns between the wavenumber range (2000 - 800 cm^-1^) of the calibration FTIR spectra (not shown). Outliers identified by the Mahalanobis distance test (8%) were removed prior to calibration and cross validation.

**Figure 3:**
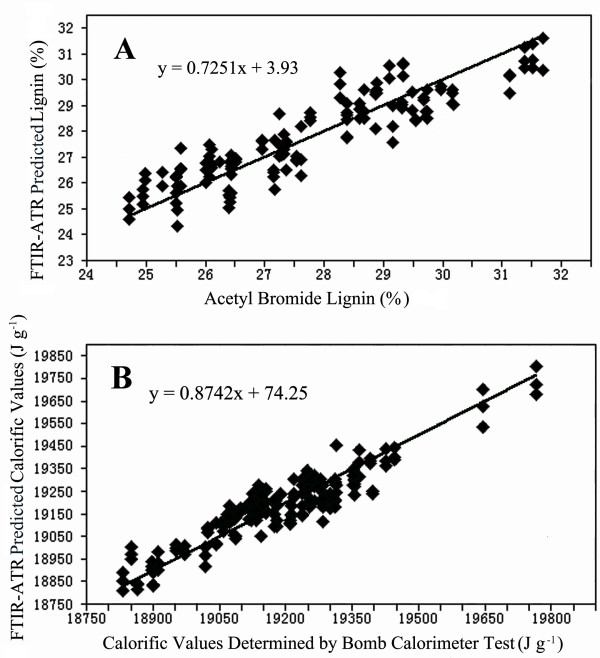
**Partial least square regression (PLSR) models for the prediction of lignin (A) and energy (B) content in extractive-free poplar wood exhibiting a natural range of variability**. Lignin was measured with the acetyl bromide method. The energy content was determined with a combustion calorimeter. Plots of measured versus predicted values for lignin (**A**) and energy (**B**) content were calculated with the best models with cross-validation results after data preprocessing and automatic wavenumber selection (Table 1 and Table 2). Solid lines represent regression line of best fit between measured and predicted values.

The FTIR spectra were, thus, suitable to construct predictive models for lignin and the energy contents, respectively. To optimize the model, several data pre-processing methods were examined for the wavenumber range from 2000 - 700 cm^-1 ^and for selected wavenumbers, respectively. With respect to lignin, vector normalization improved the calibration model [R^2 ^= 0.782 and a low number of PLS factors (4)] in comparison with the utilization of raw spectra when the spectral range between 2000 and 700 cm^-1 ^was included (Table 1). Application of this spectral range implies that the exclusion of wavenumbers unrelated to functional groups within the lignin molecules would not reduce the predictive ability of the PLS models. To test this assumption, the wavenumber range between 1650 cm^-1 ^and 1380 cm^-1^, which exhibited the largest differences for samples differing in lignin (Figure 2), was selected for model construction. The resultant predictive calibration was significantly improved because the R^2 ^values for the model statistics increased from 0.782 to 0.823 for calibration, and from 0.666 to 0.734 for cross validation (Table 1). Correspondingly, root mean square errors also decreased for both calibration and cross validation (Table 1). This supports that the observed differences in the FTIR-ATR spectra of different trees were associated with changes in the lignin contents and indicates that inclusion of unrelated wavenumbers in the model construction decreases the predictive power. We, therefore, also tested automatic wavenumber selection for the prediction of lignin content applying a set of pre-defined frequency regions and combinations of subregions. This method achieved the best test statistics based on high R^2 ^and low values for RMSEC and RMSEP; automatic wavenumber selection yielded the following reduced wavenumber ranges: 1802 - 1690 cm^-1^, 1362 - 1250 cm^-1^, and 1140 - 1028 cm^-1^. The predictive model for lignin content constructed on this basis including the first 12 of the total of 16 calculated PLS factors and accounted for 90.6% of the variance in the predicted lignin content values. The inclusion of the subsequent 4 PLS factors caused merely marginal increases (92.4%) and was therefore, not taken into account. The FTIR-ATR prediction model resulting from internal cross validation for lignin was highly acceptable as shown by the plot of the measured versus the predicted lignin contents (Figure 3A). This prediction model did not include wavenumber ranges typical for lignin, and the number of PLS factors used in the model is relatively high compared to the other models.

**Table 1:** Performance indicators of Fourier transform infrared spectroscopy with attenuated total reflection-based partial least squares regression model for prediction of acetyl bromide lignin content within lignin ranges from 23.4% to 32.1% for calibration and from 23.6% to 29.9% for independent validation, with various preprocessing methods as well as manual and automatic restriction of the wavenumber range

	Mid-infrared region ^b^				Automatic restriction ^c^	Manual restriction ^d^
	
Descriptor ^a^	No preprocessing	1^st ^derivative	VN	1^st ^derivative + VN	VN	VN
R^2 ^(calibration)	0.770	0.742	0.782	0.778	0.906	0.823
R^2 ^(cross-validation)	0.614	0.638	0.666	0.651	0.806	0.734
RMSEC (%)	0.894	0.940	0.864	0.876	0.584	0.770
RMSECV (%)	0.937	0.978	0.940	0.952	0.743	0.860
RMSEP (%)	1.13	1.09	1.05	1.07	0.80	0.91
No. of PLS factors	6	4	4	5	12	3

^a ^Descriptor explanations are as follows: R^2 ^= coefficient of determination (a measure of the degree of fit of the regression); RMSEC = root mean square error of the calibration samples; RMSECV = root mean square error of the cross validation samples; RMSEP = root mean square error of the prediction samples; VN = vector normalization.

^b ^Wavenumber range for the mid-infrared region is 2000 - 700 cm^-1^.

^c ^The wavenumber ranges resulting from automatic restriction are 1802 - 1690 cm^-1^, 1362 - 1250 cm^-1^, and 1140 - 1028 cm^-1^.

^d ^The wavenumbers resulting from manual restriction are 1650 - 1380 cm^-1^.

Concerning the estimation of energy content, data pre-processing (i.e., first derivative and baseline correction with Rubberband method) also led to an improved calibration model with a high R^2 ^(0.865) but a relatively high number of PLS factors (10) (Table 2). The model was slightly improved by automatic wavenumber selection with pre-defined frequency regions and combinations of subregions, which reduced the range from 2000 to 700 cm^-1 ^to the finger print region of 1770 - 990 cm^-1^. The predictive model for energy content with automatic wavenumber selection including the first 11 of 16 total calculated PLS factors was considered most appropriate, as this model accounted for 87.6% of variance in the predicted calorific values (Table 2, Figure 3B).

**Table 2:** Performance indicators of Fourier transform infrared spectroscopy with attenuated total reflection-based partial least squares regression model for predication of gross calorific values within a range from 19767 to 17260 J g^-1 ^for the calibration set and from 18255 to 17345 J g^-1 ^for the external validation set with various preprocessing methods as well as automatic restriction of the wavenumber range

Descriptor ^a^	Mid-infrared region ^b^				Automatic restriction ^c^
	
	No preprocessing	1^st ^derivative	BLC	1^st ^derivative + BLC	BLC
R^2 ^(calibration)	0.856	0.873	0.869	0.874	0.904
R^2 ^(cross-validation)	0.646	0.662	0.697	0.686	0.787
RMSEC (J g^-1^)	67.5	66.1	66.4	65.7	62.1
RMSECV (J g^-1^)	99.8	92.0	86.2	84.1	76.7
RMSEP (J g^-1^)	104	103	99	94	87.3
No. of PLS factors	12	10	11	10	11

^a ^Descriptor explanations are as follows: R^2 ^= coefficient of determination (a measure of the degree of fit of the regression); RMSEC = root mean square error of the calibration samples; RMSECV = root mean square error of the cross validation samples; RMSEP = root mean square error of the prediction samples; BLC = Baseline correction, Rubberband method.

^b ^The wavenumbers for the mid-infrared region are 2000 - 700 cm^-1^.

^c ^The wavenumber range resulting from automatic restriction is 1770 - 990 cm^-1^.

The performances of the FTIR-based predictive models for the lignin content were comparable to other studies employing NIRS or FTIR spectroscopy [5,8,11,21,22,41,42]. In those previous studies R^2 ^values for the lignin models ranged from 0.74 to 0.98 and for the independent validation from 0.57 to 0.97, respectively and the corresponding errors RMSEC and RMSECV were 0.58 to 1.0% and 0.36 to 1.6%, respectively. However, caution must be exercised when comparing the regression coefficients because many variables such as the spectral range [5,8], utilization of raw spectra [22] or smoothing, offset and normalization [21], and tree species [41,42] used for the predictive models may all have significant effects on this parameter. Although some previous studies obtained slightly stronger correlations and lower errors our predictive models are still sufficiently strong to cope with the relatively limited ranges of lignin and energy contents arising from natural intra-specific variability.

### External validation of the optimized calibration model for the estimation of lignin and energy content

**Figure 4:**
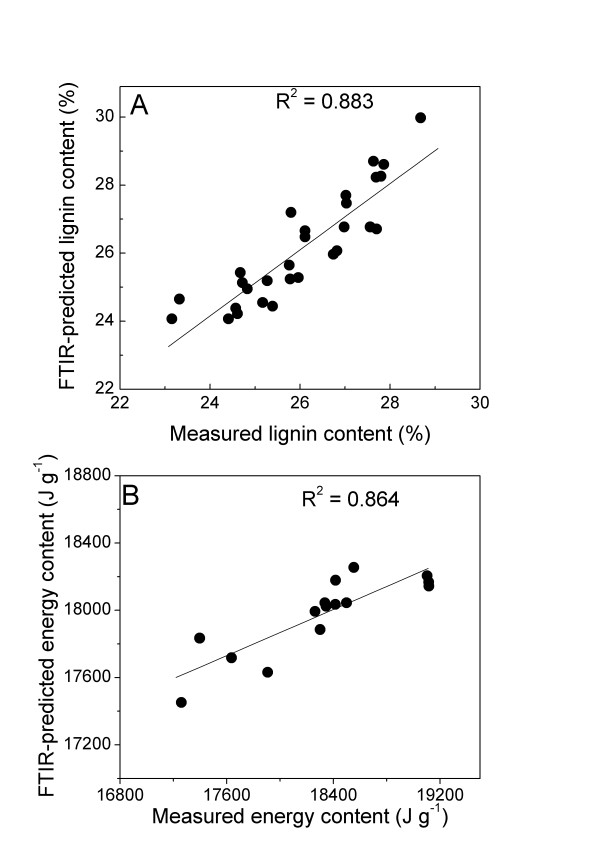
**External validation of the PLSR models for lignin (A) and energy contents (B) prediction**. FTIR-ATR spectra were produced for an independent set of wood samples and used to predict the lignin or energy contents using the best models from table 1 and 2, respectively. Lignin and energy contents were determined by the acetyl bromide method and a combustion calorimeter, respectively. The predicted values were plotted against the measured values. Solid line represents regression line of best fit between measured and predicted values.

External validation was performed using the best predictive model obtained after internal cross validation. For this purpose an additional set of independent wood samples was scanned and the FTIR-ATR spectra were used to predict the amount of lignin and energy contents, respectively. Samples whose predicted values exceeded the calibration range were counted as outliers and excluded from the evaluation procedure. The samples were also used for the determination of lignin and energy contents in the evaluation step. The predicted values were plotted as dependent and the measured values as independent variables. The regression models for the validation of lignin and energy content gave high R^2 ^values and low root mean square errors of prediction (RMSEP = 0.75% and 69 J g^-1 ^respectively, Figures 4A and 4B). In general, the predicted values for independent validation samples were in good agreement with experimental data, even though for the validation of energy content wood material from different growth conditions was used.

## Conclusions

In this study we have shown that the natural variation of wood components in extractive-free samples of hybrid poplar wood was sufficient for the construction of calibration models for lignin and the energy contents. This required the determination of lignin via wet chemical methods and the measurement of calorific values through calorimetry, acquisition of high quality FTIR spectra achieved by the application of an ATR unit and the building PLS model by means of a chemometric software. Once established, data acquisition time for the analysis of extracted wood materials is reduced to minutes and permits large numbers of samples to be processed. The optimized and externally validated calibrations are of sufficient quality to efficiently assess lignin and energy content of poplar wood in large-scale breeding or genetic engineering programmes. FTIR-ATR spectroscopy in combination with partial least squares regression modelling may also be useful for the optimization of wood utilization in the pulping industry or for biofuel or heat production purposes. Furthermore, our study documents for the first time that the intra-specific variability of lignin and energy contents are unrelated to each other. Using principle component analyses we identified influential wavenumbers for lignin and energy contents. While the factor loadings for lignin identified as expected aromatic compounds, carbohydrate ring vibrations were prevalent for the energy content. As both traits are apparently associated with different chemical constituents, we suggest that it will be possible to improve the energy content of wood without a concomitant increase in lignin.

## Methods

### Plant material and sample preparation

Single stem hybrid poplars (*Populus trichocarpa × deltoides*) were coppiced and plant material was harvested in the fourth year of the second coppice cycle (n = 95 individuals) on the field site at Headley (U.K.). Further details of the plantation have been described elsewhere [4]. The stems were stripped of bark and pith. Wood blocks were oven-dried (60 °C) for 2 days. For some validation experiments one-year-old poplar wood (*P*. × *canescens*), grown in Göttingen (Germany), was used (n = 15 individuals). Dry wood was cut into small pieces with secateurs and ground to a flour in a ball mill (MM2000, Retsch, Haan, Germany) at an amplitude of 90 min^-1 ^for approximately 4 min in liquid nitrogen to prevent heating and to accelerate the milling process. A fine powder with a particle size less than 20 μm was achieved to avoid disturbance originating from the influence of particle size on FTIR spectra [40].

Interfering extraneous substances (e.g., soluble fats, waxes, simple sugars, and low-molecular soluble phenolics) were removed by extraction with acetone. For this purpose the wood mill was successively extracted 4-times for 2-days in 100% acetone at room temperature [43]. The resulting extractive-free wood, also known as structural biomass or plant cell walls, was used for all further analyses.

### Wet chemical analysis of lignin content

The lignin content of wood powder was determined using a modified acetyl bromide method [44]. One mL of freshly prepared 25% (w/w) acetyl bromide/glacial acetic acid solution was added to 1 mg air-dry, extractive-free wood powder in a 2-mL polypropylene safe-seal micro-tube (Sarstedt, Nümbrecht, Germany). The micro-tube was sealed, placed in a water bath and maintained for 30 min with repeated mixing at 70 °C. Subsequently, the reaction was stopped by cooling the micro-tube in an ice-water bath. The reaction mixture was mixed and 100 μL of the mixture was transferred into a 2 mL safe-seal micro-tube containing 200 μL of 2.0 M sodium hydroxide. The volume was made up to 2 mL with 1.7 mL of glacial acetic acid. The UV absorbance of the solution was determined at 280 nm against a blank solution which was run in conjunction with the sample. The extinction coefficient of lignin extracted by acetyl bromide of ε = 20.09 L·g^-1^·cm^-1 ^was used to calculate the lignin contents of the samples [44]. All analyses were conducted in triplicate and means were calculated for each of the 95 wood samples from Headley. The pooled standard deviation obtained by the assay was 0.042%. The lignin content was expressed as percentage of oven-dry extractive-free wood. Moisture content of the wood powder was determined before and after drying at 60 °C.

### Determination of the calorific value of wood

The calorific value of the extractive-free wood was analyzed with a bomb calorimeter (IKA^® ^C200 Calorimeter System; IKA^® ^Werke GmbH & Co. KG, Staufen, Germany). About 100 mg extractive-free wood was weighed and pressed into a pellet using a press attached to the calorimeter. The resultant pellet placed inside a combustible crucible was then combusted with O_2 _(ca. 30 mbar) in a decomposition bomb. The calorific value was determined as the increase in the temperature of the water as a direct measure for the internal energy of the burning reaction in the decomposition vessel via an isoperibolic automatic procedure. Benzoic acid tablets were used as the standard (net calorific value: 26457 ± 20 J g^-1^) to calculate the calorific values of the samples. All tests were performed in duplicate and means were calculated for 61 samples from Headley and 15 samples from Göttingen. It was not possible to use all 95 Headley samples because we did not have sufficient material.

### FTIR-ATR spectroscopy

The FTIR-ATR spectra of extractive-free wood powder were measured with the FTIR spectrometer Equinox 55 (Bruker Optics, Ettlingen, Germany), equipped with a deuterium triglycine sulfate detector and an attached ATR unit (DuraSamplIR, SensIR Europe, Warrington, UK). The scanning range was from 600 to 4000 cm^-1 ^with a resolution of 4 cm^-1^.

The wood powder was pressed against the diamond crystal of the ATR device. A pressure applicator with a torque knob ensured that the same pressure was applied for all measurements. For each wood sample, 32 scans were acquired and averaged. Background scanning and correction was carried out regularly at 15-20 min intervals. For each sample, three different subsamples were measured and the resultant mean spectra were used for further analyses. The standard deviation of spectra of the subsamples was obtained by the OPUS 5.5 software (Bruker Optics, http://www.brukeroptics.com/). The standard deviations of the different biological samples were used to create an overall standard deviation using the multi-evaluation tool in the OPUS software. All samples were included.

### Principal component analysis (PCA) of selected FTIR spectra

For PCA we identified two sample sets, each consisting of 30 spectra. One contained 10 wood samples with lowest, 10 with medium and 10 with the highest lignin content measured with the acetyl bromide method in 95 Headley samples. The second set consisted of 10 wood samples with the lowest, 10 with medium and 10 with the highest calorific values determined with the bomb calorimeter in 61 Headley samples. For data analysis, the region of 1800-900 cm^-1 ^of the FTIR spectra was baseline-corrected via the Rubberband method, vector-normalized, and mean-centred. Then the data were used for PCA. PCA removes the redundancy of having many data points varying in a correlated way by transforming the original data into a set of new and uncorrelated PCs. The first four factor loadings were plotted to gather information about the major components responsible for variability in the fingerprint region of the IR spectrum. All mathematical operations were carried out with OPUS version 5.5 software (Bruker Optics, http://www.brukeroptics.com/).

### Calibration development and statistical analysis

The calibration models were developed using the QUANT 2 chemometric software package provided in the OPUS 5.5 software (Bruker Optics, http://www.brukeroptics.com/). For calibration and internal validation of lignin contents, the 95 Headley samples were split into two groups, a first group of 61 samples for internal validation (calibration and cross validation) and a second group of 34 samples for external validation of lignin.

For the calibration and validation of calorific values, aliquots of the same group of 61 samples from Headley employed for lignin analyses and additionally 15 samples from Göttingen were used. Of this set 15 samples (some from Göttingen and some from Headley) were removed as independent validation set. The remaining samples were used for calibration and cross validation. Subsequently, the model was validated by testing the validation.

For all calibrations, the following data pre-processing algorithms were tested prior to model construction: first derivative, vector normalization, baseline correction (Rubberband method), and first derivative + vector normalization. Subsequent to pre-processing, wavenumber selection was executed either by iteratively combining and restricting wavenumber ranges or by automatically choosing wavenumber ranges via a set of pre-defined frequency regions and combinations of subregions.

The QUANT software package can be used for principal component analysis (PCA) and for developing partial square models (PLS modelling). The FTIR-ATR spectra for all wood samples were combined into a single data matrix (X-matrix) and the values obtained by chemical lignin analyses or by bomb calorimetry were combined into a response matrix (Y-matrix). The calibration spectra were mean centred by subtracting the mean spectrum from each sample spectrum prior to PLS modelling. Wood component concentrations and energy contents were also mean centred. The PLS algorithm available in the QUANT software package simultaneously decomposes both absorbance spectra and constituent (or calorific value) matrices. The number of principal components (or factors) used for PLS prediction model was determined by observing the response of the residual Y-variance with added factors. When additional factors did not substantially reduce the residual Y-variance, the model constructing process was completed.

All PLS models were constructed with cross validation. The cross validation process was performed as follows: One sample was removed systematically from the data set, then a PLS mode was constructed with the remaining samples to predict the value of the Y-variable for the removed sample. This process continued until each sample had been excluded from the data set and used for validation.

### External validation

External validation of the PLS model for the estimation of lignin content was performed with an independent sample set consisting of 34 hybrid poplar coppice wood samples that were not included in the development of the calibration model. External validation of the PLS model for the prediction of calorific energy value was carried out with another sample set consisting of 15 samples of stem wood from poplars that had been grown in Göttingen (Germany) or Headley (U.K.) not included in model development or cross-calibration.

### Outlier detection

Spectral outliers during multivariate calibration and validation phase were detected trough Mahalanobis distance calculations. The Mahalanobis distance is a measure of the similarity of the analyzed spectrum and the mean value of all others [45]. A spectrum with a Mahalanobis distance larger than the limit [Limit = (Factor × Rank)/M; M is the number of samples in the calibration dataset] can be recognized as an outlier and removed from the list of standards.

The factor ranged between 2 and 10. To calculate the limit of the Mahalanobis distance, a factor of two was too restrictive for the prediction of unknown natural samples. As a consequence, too many samples were marked as outliers. A factor of five was used in this study. 8% of the calibration samples were detected as outliers in calibration and cross validation stages.

## Authors' contributions

GZ performed the experiments and wrote the manuscript. GT supplied material and participated in the preparation of the manuscript. AP conceived the project, supervised the experiments and preparation of the manuscript. All authors read and approved the final manuscript.

## Competing interests

The authors declare that they have no competing interests.

## Supplementary Material

Additional file 1 **Figure S1 - Factor loadings of base-line corrected and normalized mean spectra of poplar wood samples exhibiting natural variability in energy and lignin contents**. (a) first, (b) second, (c) third, and (d) fourth factor loadings for lignin (black line) and energy content (red line), respectively. The different numbers in the figures refer to absorption peaks described in Additional file 2, Table S1 and Additional file 3, Table S2. According to the approach of Tillmann [46], Rana et al. [47] and Nuopponen et al. [41] the first eight highest peaks were assigned in the factor loading for PC1 (a), PC2 (b), PC3 (c), and PC4 (d), respectively. PC1, PC2, PC3, and PC4 of the "lignin set" (black line) explained 68.7%, 22.2%, 6.8%, and 2.3% of the variation respectively. PC1, PC2, PC3, and PC4 in the "energy sample set" (red line) explained 73.3%, 20.1%, 5.5%, and 1.1% of the variation, respectively. Click here for file

Additional file 2 **Table S1 - Absorption band assignments of the first (PC1), second (PC2), third (PC3) and fourth (PC4) factor loadings obtained by principal component analysis for the energy content**. The eight highest peaks are indicated for each factor loading. The numbers in parentheses indicate the position according to peak height (see Additional file 1, Figure S1). Click here for file

Additional file 3 **Table S2 - Absorption band assignments of the first (PC1), second (PC2), third (PC3) and fourth (PC4) factor loadings obtained by principal component analysis for lignin content**. The eight highest peaks are indicated for each factor loading (Additional file 1, Figure S1). The numbers in parentheses indicate the position according to peak height. Click here for file
