# Supplementary material for: FTIR-ATR-based prediction and modelling of lignin and energy contents reveals independent intra-specific variation of these traits in bioenergy poplars
Source: Plant Methods. 2011 Apr 10;7:9. doi: 10.1186/1746-4811-7-9 (PMC3094334; doi:10.1186/1746-4811-7-9)
Supplement: Additional file 2 — Table S1 - Absorption band assignments of the first (PC1), second (PC2), third (PC3) and fourth (PC4) factor loadings obtained by principal component analysis for the energy content. The eight highest peaks are indicated for each factor loading. The numbers in parentheses indicate the position according to peak height (see Additional file 1, Figure S1). [file 1746-4811-7-9-S2.DOC]

Additional Table S1

Absorption band assignments of the first (PC1), second (PC2), third (PC3) and fourth (PC4) factor loadings obtained by principal component analysis for the energy content. The eight highest peaks are indicated for each factor loading. The numbers in parentheses indicate the position according to peak height (see Additional Figure S1).

|  | Wavenumber (cm^-1^) | | | Band origin | References |
| --- | --- | --- | --- | --- | --- |
| 1st  factor | 2nd factor | 3rd factor | 4^th^ factor |
| 1026 (1) |  |  |  | C-H in G lignin, C-O deformation in prim. alcohols | Usmanov et al. [1] |
| 1039 (2) |  |  |  | C-O vibration in cellulose and hemicelluloses | Hergert [2] |
| 1066 (3) |  |  |  | C-H, C-O deformations | Fengel and Wegener [3] |
| 1576 (4) |  |  |  | No information available |  |
| 1525 (5) |  |  |  | No information available |  |
| 1473 (6) |  |  |  | No information available |  |
| 1714 (7) |  |  |  | C=O from xylan | Schwanninger [4] |
| 1267 (8) |  |  |  | C-O stretching of lignin and xylan | Socrates [5] |
|  | 989 (1) |  |  | -HC=CH- out-of-plane deformation | Faix [6] |
|  | 1071 (2) |  |  | C-O deformation in secondary alcohols and alphatic ethers | Faix [6] |
|  | 1045 (3) |  |  | C-O-C, C-O deformation by ring vibration of carbohydrates | Faix et al. [7], Naumann et al. [8] |
|  | 1128 (4) |  |  | C-O-C, C-O dominated by ring vibration of carbohydrates | Faix et al. [7], Naumann et al. [8] |
|  | 1653 (5) |  |  | Adsorbed O-H and conjugated C-O | Pandey [9] |
|  | 1607 (6) |  |  | Aromatic skeletal vibration in lignin | Pandey [9] |
|  | 1267 (7) |  |  | C-O stretching of lignin and xylan | Socrates [5] |
|  | 1508 (8) |  |  | Aromatic skeletal vibration in lignin | Pandey [9] |
|  |  | 1033 (1) |  | Aromatic C-H in plane deformation, guaiacyl type and C-O deformation, primary alcohol | Faix [6], Hergert [2] |
|  |  | 1051 (2) |  | C-H, C-O deformations | Fengel and Wegener [3] |
|  |  | 1747 (3) |  | Unconjugated C=O stretch in xylans (hemicelluloses) from acetates (acetic acid esters) | Fengel and Wegener [3] |
|  |  | 989 (4) |  | -HC=CH- out-of-plane deformation | Faix [6] |
|  |  | 1620 (5) |  | Aromatic skeletal vibration in lignin | Naumann et al. [8] |
|  |  | 1406 (6) |  | C-O stretching | Faix [6], Hergert [2] |
|  |  | 1232 (7) |  | Syringyl ring and C=O stretch in lignin and xylans | Socrates [5] |
|  |  | 1084 (8) |  | Asymmetric stretching of C-O-C in esters | Schwanninger [4]; Faix [6] |
|  |  |  | 1229 (1) | Syringyl ring and C=O stretch in lignin and xylans | Socrates [5] |
|  |  |  | 1738 (2) | Unconjugated C=O stretch in xylans (hemicelluloses) from acetates (acetic acid esters) | Fengel and Wegener [3] |
|  |  |  | 1057 (3) | C-H, C-O deformations | Fengel and Wegener [3] |
|  |  |  | 1105 (4) | C-O-C, C-O dominated by ring vibration of carbohydrates | Faix et al. [7], Naumann et al. [8] |
|  |  |  | 1030 (5) | Same as peak No. 4 | Faix et al. [5], Naumann et al. [6] |
|  |  |  | 1541 (6) | C-H deformations; asymmetrical in –CH_3_ and –CH_2_ | Schwanninger [4]; Socrates [5] |
|  |  |  | 1655 (7) | Adsorbed O-H and conjugated C-O | Pandey [9] |
|  |  |  | 1163 (8) | Typical for lignin; C=O in ester groups | Socrates [5] |
