# Supplementary material for: FTIR-ATR-based prediction and modelling of lignin and energy contents reveals independent intra-specific variation of these traits in bioenergy poplars
Source: Plant Methods. 2011 Apr 10;7:9. doi: 10.1186/1746-4811-7-9 (PMC3094334; doi:10.1186/1746-4811-7-9)
Supplement: Additional file 3 — Table S2 - Absorption band assignments of the first (PC1), second (PC2), third (PC3) and fourth (PC4) factor loadings obtained by principal component analysis for lignin content. The eight highest peaks are indicated for each factor loading (Additional file 1, Figure S1). The numbers in parentheses indicate the position according to peak height. [file 1746-4811-7-9-S3.DOC]

Additional Table S2

Absorption band assignments of the first (PC1), second (PC2), third (PC3) and fourth (PC4) factor loadings obtained by principal component analysis for lignin content. The eight highest peaks are indicated for each factor loading (Additional Figure S1). The numbers in parentheses indicate the position according to peak height.

| Wavenumber (cm^-1^) | | | | Band origin | References |
| --- | --- | --- | --- | --- | --- |
| 1st  Factor | 2nd factor | 3rd factor | 4th factor |
| 1025 (1) |  |  |  | C-H in G lignin, C-O deformation in prim. alcohols | Usmanov et al. [1] |
| 1037 (2) |  |  |  | C-O vibration in cellulose and hemicelluloses | Hergert [2] |
| 1064 (3) |  |  |  | C-H, C-O deformations | Fengel and Wegener [3] |
| 1294 (4) |  |  |  | Amide I and II in protein (upward direction) | Naumann et al. [8] |
| 1148 (5) |  |  |  | C-H in plane deformation of syringyl rings | Harrington et al. [10] |
| 1688 (6) |  |  |  | C=O in lignin | Harrington et al. [10] |
| 1587 (7) |  |  |  | No information available |  |
| 1120 (8) |  |  |  | Aromatic skeletal and C-O stretch | Pandey and Pitman [11] |
|  | 1032 (1) |  |  | Aromatic C-H in plane deformation, guaiacyl type and C-O deformation, primary alcohol | Faix [6], Hergert [2] |
|  | 993 (2) |  |  | C-O-C, C-O deformation by ring vibration of carbohydrates | Faix et al. [7], Naumann et al. [8] |
|  | 1092 (3) |  |  | Asymmetric stretching of C-O-C in esters | Schwanninger [4]; Faix [6] |
|  | 1067 (4) |  |  | C-H, C-O deformations | Fengel and Wegener [3] |
|  | 1126 (5) |  |  | Aromatic skeletal and C-O stretch | Pandy and Pitman [11] |
|  | 1248 (6) |  |  | Syringyl nuclei deformation combined with deformation of cellulose | Evans [12] |
|  | 1207 (7) |  |  | Syringyl ring and C=O stretch in lignin and xylans | Socrates [5] |
|  | 1335 (8) |  |  | S ring plus G ring condensed | Faix [6], Hergert [2] |
|  |  | 1207 (1) |  | Syringyl ring and C=O stretch in lignin and xylans | Socrates [5] |
|  |  | 1263 (2) |  | G ring plus C=O stretch | Faix [6], Evans [12] |
|  |  | 1072 (3) |  | C-O deformation in secondary alcohols and aliphatic ethers | Faix [6] |
|  |  | 1719 (4) |  | C=O stretch in unconjugated ketones, carbonyls and in ester groups (frequently of carbohydrate origin) | Faix [6], Pandey and Pitman [11] |
|  |  | 1759 (5) |  | Same as peak No. 4 | Faix [6], Pandey and Pitman [11] |
|  |  | 984 (6) |  | -HC=CH- out-of-plane deformation | Faix [6] |
|  |  | 1120 (7) |  | Aromatic skeletal and C-O stretch | Pandey and Pitman [11] |
|  |  | 1578 (8) |  | Asymmetrical deformation of NH_3_^+^ | Socrates [5] |
|  |  |  | 1680 (1) | No information available |  |
|  |  |  | 1580 (2) | Asymmetrical deformation of NH_3_^+^ | Socrates [5] |
|  |  |  | 1049 (3) | C-O-C, C-O deformation by ring vibration of carbohydrates | Faix et al. [7], Naumann et al. [8] |
|  |  |  | 1126 (4) | Aromatic skeletal and C-O stretch | Pandey and Pitman [11] |
|  |  |  | 1020 (5) | C=O stretch from lignin | Schwanninger [4] |
|  |  |  | 947 (6) | C-O-C, C-O dominated by ring vibration of carbohydrates | Faix et al. [7], Naumann et al. [8] |
|  |  |  | 1163 (7) | Typical for HGS lignins; C=O in ester groups | Faix [6] |
|  |  |  | 1259 (8) | G ring plus C=O stretch | Faix [6], Evans [12] |

References to Additional files

1. Usmanov KU, Yulchibaev AA, Dordzhin GS, Valiev A: **Ir spectroscopic analysis of graft co-polymers of cellulose and its derivatives with vinyl fluoride.** *Fibre Chem* 1972, **3:**292-295.

2. Hergert HL: **Infrared spectra.** In *Lignins: Occurrence, Formation, Structure and Reactions.* Edited by Sarkanen KV, Ludwig CH. New York: Wiley Interscience; 1971: 267-297

3. Fengel D, Wegener G: *Wood - Chemistry, Ultrastructure, Reactions.* Berlin: Walter De Gruyter Inc; 1989.

4. Schwanninger M, Rodrigues JC, Pereira H, Hinterstoisser B: **Effects of short-time vibratory ball milling on the shape of FT-IR spectra of wood and cellulose.** *Vibrational Spectroscopy* 2004, **36:**23-40.

5. Socrates G: *Infrared and Raman characteristic group frequencies: tables and charts.* 3rd edn. West Sussex: John Wiley & Sons Ltd.; 2004.

6. Faix O: **Classification of lignins from different botanical origins by FTIR spectroscopy.** *Holzforschung* 1991, **45:**21-27.

7. Faix O, Bremer J, Schmidt O, Tatjana SJ: **Monitoring of chemical changes in white-rot degraded beech wood by pyrolysis - gas chromatography and Fourier-transform infrared spectroscopy.** *J Anal Appl Pyrol* 1991, **21:**147-162.

8. Naumann D, Helm D, Labischinski H, Giesbrecht P: **The characterization of microorganisms by Fourier transform infrared spectroscopy (FTIR).** In *Modern Techniques for Rapid Microbiological Analysis.* Edited by Nelson WH. New York: Wiley-VCH; 1991: 43-96

9. Pandey KK: **A study of chemical structure of soft and hardwood and wood polymers by FTIR spectroscopy.** *J Appl Polym Sci* 1999, **71:**1969-1975.

10. Harrington KJ, Higgins HG, Michell AJ: **Infrared Spectra of Eucalyptus regnans F. Muell. and Pinus radiata D. Don.** *Holzforschung* 1964, **18:**108-113.

11. Pandey KK, Pitman AJ: **FTIR studies of the changes in wood chemistry following decay by brown-rot and white-rot fungi.** *Int Biodeter & Biodegr* 2003, **52:**151-160.

12. Evans PA: **Differentiating "hard" from "soft" woods using Fourier transform infrared and Fourier transform spectroscopy.** *Spectrochim Acta A* 1991, **47:**1441-1447.
